# Now’s the Time

**Published:** 1888-04

**Authors:** 


### NOW'S THE TIME.

The coming meeting at Galveston will be a fitting time for members and visiting physicians to testify their appreciation of the effort of the *Journal* in the interest of organization, and the general welfare of the profession, by coming forward and subscribing for Volume 4. Volume 4 will begin July, 1st, under most favorable auspices, and the *Journal* will continue to be, as in the past, the exponent of rational medicine in Texas, and the repository of all the current medical news of the day. No member of the State Association *can afford to do without it*, if he would keep up with the progress of medicine in the Lone Star State, and know what our own State Association and all the local societies are doing; for *The Journal* will publish all official documents and announcements; will be the mouthpiece of the Association, speaking to the members and to the world. We have even had letters from physicians, (non-subscribers of course) asking "when and where the next meeting of the State Association will be!"

The *Journal* takes this occasion to return its most cordial acknowledgement to the liberal and enlightened members of the profession, who have so nobly and so effectually rendered their support, moral and pecuniary, whereby its brilliant success has been attained. The *Journal* is universally pronounced *a credit to Texas*, and has taken and maintained a leading position in the ranks of current medical literature. Texas *should be* proud of it, as its friends and supporters are, and we hope, at the coming meeting to enroll the name of every enlightened and liberal minded member in attendane, as a friend and supporter of *The Texas Medical Journal.*

Several hundred copies of this handsome edition will be distributed gratuitously, at the Galveston meeting, in order that all may see and examine it before subscribing. Every lover of legitimate medicine, and advocate of progress in the science, and of organization as a means to ends, should promptly subscribe for his home journal.

